# Author Correction: Sex-specific innate immune selection of HIV-1 in utero is associated with increased female susceptibility to infection

**DOI:** 10.1038/s41467-020-16215-7

**Published:** 2020-05-04

**Authors:** Emily Adland, Jane Millar, Nomonde Bengu, Maximilian Muenchhoff, Rowena Fillis, Kenneth Sprenger, Vuyokasi Ntlantsana, Julia Roider, Vinicius Vieira, Katya Govender, John Adamson, Nelisiwe Nxele, Christina Ochsenbauer, John Kappes, Luisa Mori, Jeroen van Lobenstein, Yeney Graza, Kogielambal Chinniah, Constant Kapongo, Roopesh Bhoola, Malini Krishna, Philippa C. Matthews, Ruth Penya Poderos, Marta Colomer Lluch, Maria C. Puertas, Julia G. Prado, Neil McKerrow, Moherndran Archary, Thumbi Ndung’u, Andreas Groll, Pieter Jooste, Javier Martinez-Picado, Marcus Altfeld, Philip Goulder

**Affiliations:** 1 Department of Paediatrics, University of Oxford, Oxford, UK; 2 HIV Pathogenesis Programme, The Doris Duke Medical Research Institute, University of KwaZulu-Natal, Durban, South Africa; 3 Umkhuseli Innovation and Research Management, Pietermaritzburg, South Africa; 4 Max von Pettenkofer Institute, Virology, National Reference Center for Retroviruses, Faculty of Medicine, LMU München, Munich, Germany; 5 German Center for Infection Research (DZIF), Partner site Munich, Munich, Germany; 6 Department of Medicine, University of KwaZulu-Natal, Durban, South Africa; 7 Department of Infectious Diseases, Ludwig-Maximilians-University, Munich, Germany; 8 Africa Health Research Institute, Durban, South Africa; 9 Department of Medicine, University of Alabama at Birmingham, Birmingham, AL USA; 10 Birmingham Veterans Affairs Medical Center, Research Service, Birmingham, AL 35233 USA; 11 Stanger Hospital, KwaDukuza, KwaZulu-Natal, South Africa; 12 KwaZulu-Natal Department of Health, Pietermartizburg, South Africa; 13 Mahatma Gandhi Memorial Hospital, Phoenix, KwaZulu-Natal South Africa; 14 Queen Nandi Regional Hospital, Empangeni, KwaZulu-Natal South Africa; 15 Edendale Hospital, Pietermartizburg, KwaZulu-Natal, South Africa; 16 Nuffield Department of Medicine, University of Oxford, Oxford, UK; 17 IrsiCaixa AIDS Research Institute, Germans Trias i Pujol Research Institute (IGTP), Badalona, Spain; 18 Department of Paediatrics, University of KwaZulu-Natal, Durban, South Africa; 19 Ragon Institute of MGH, MIT, and Harvard, Cambridge, Massachusetts, USA; 20 TU Dortmund University, Faculty of Statistics, Vogelpothsweg 87, 44227 Dortmund, Germany; 21 Department of Paediatrics, Kimberley Hospital, Northern Cape, South Africa; 22 University of Vic-Central University of Catalonia (UVic-UCC), Catalonia, Spain; 23 Institució Catalana de Recerca i Estudis Avançats (ICREA), Barcelona, Spain; 24 Virus Immunology Unit, Heinrich-Pette-Institut, Hamburg, Germany

**Keywords:** Interferons, HIV infections, Innate immunity, Translational research

Correction to: *Nature Communications* 10.1038/s41467-020-15632-y, published online 14 April 2020.

The original version of this Article contained an error in the spelling of the author Maria C. Puertas, which was incorrectly given as Mari Carmen Puertas. This has now been corrected in both the PDF and HTML versions of the Article.

