# Supplementary material for: Sex-specific innate immune selection of HIV-1 in utero is associated with increased female susceptibility to infection
Source: Nat Commun. 2020 Apr 14;11:1767. doi: 10.1038/s41467-020-15632-y (PMC7156749; doi:10.1038/s41467-020-15632-y)
Supplement: Supplementary file 1 — Supplementary Information [file 41467_2020_15632_MOESM1_ESM.pdf]

SUPPLEMENTARY INFORMATION

SEX-SPECIFIC INNATE IMMUNE SELECTION OF HIV-1 *IN-UTERO* IS ASSOCIATED WITH INCREASED FEMALE SUSCEPTIBILITY TO INFECTION

Adland and Millar et al

SUPPLEMENTARY FIGURE 1

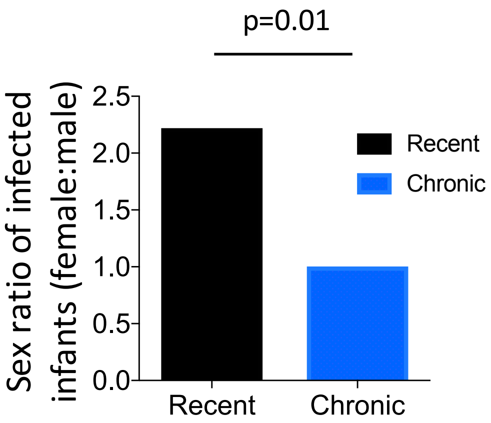

SUPPLEMENTARY FIGURE 1. Sex ratio of *in utero* infected infants categorised by timing of maternal first HIV positive test. Mothers who tested HIV positive prior to the pregnancy were categorised as 'chronic' infections and those testing positive for the first time during the pregnancy are categorised as 'recent' infections. Source data are provided as a Source Data file.

SUPPLEMENTARY FIGURE 2

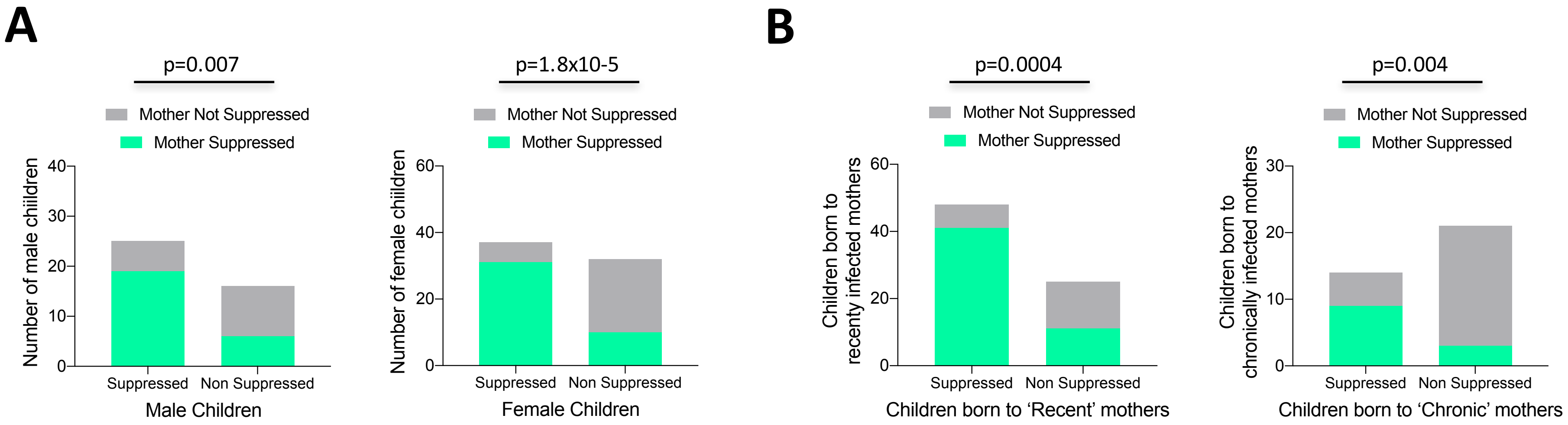

SUPPLEMENTARY FIGURE 2. A. Fisher's exact test calculations shown for suppression of viraemia (plasma RNA viral load <20 copies/ml) versus non-suppression of viraemia (plasma RNA viral load >20 copies/ml) for male children and their mothers (left hand panel) and for female children and their mothers (right hand panel). **B.** Fisher's exact test calculations shown for suppression of viraemia (plasma RNA viral load <20 copies/ml) versus non-suppression of viraemia (plasma RNA viral load >20 copies/ml) for children born to recently-infected mothers and their mothers (left hand panel) and for children of chronically-infected mothers and their mothers (right hand panel). Source data are provided as a Source Data file.

SUPPLEMENTARY FIGURE 3

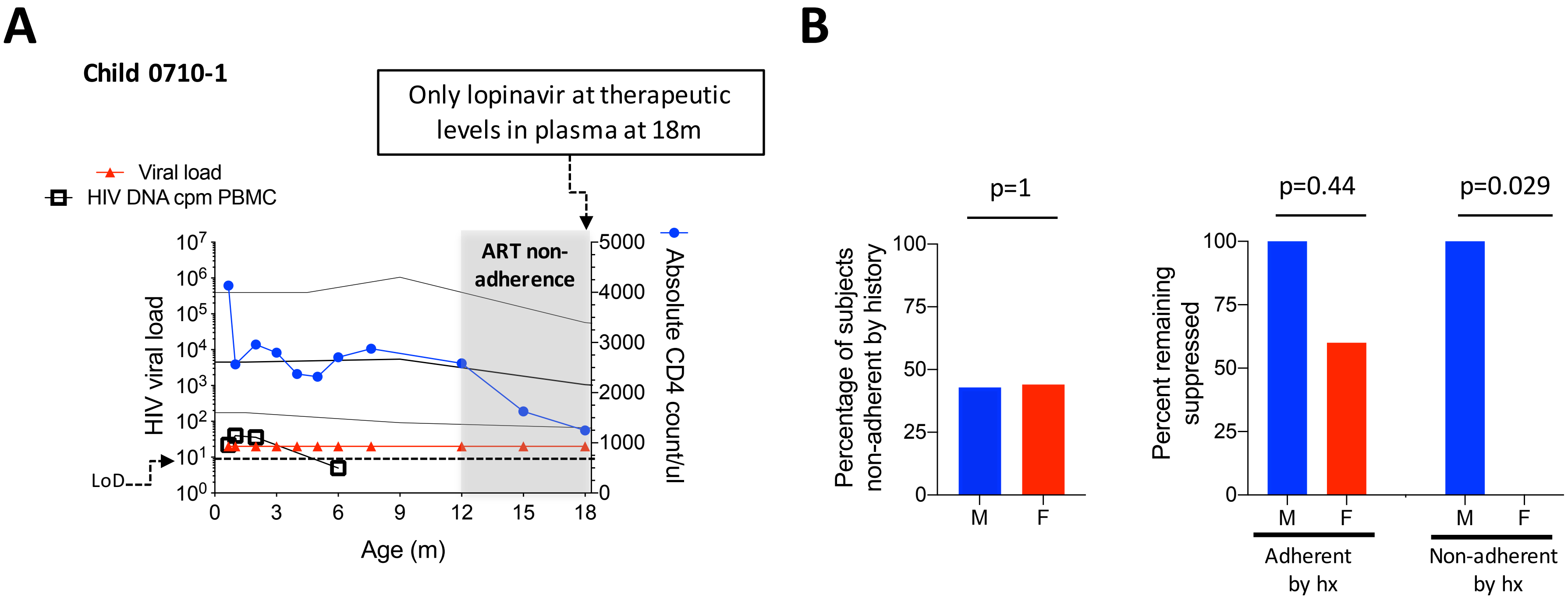

SUPPLEMENTARY FIGURE 3. Higher frequency viral rebound in females compared with males following ART non-adherence. **A.** Lack of viral rebound in a third male subject 0710-1 with an undetectable DNA viral load following a significant period of sub-optimal ART adherence. The caregiver reported missing more than 4 doses of ART medications per week (out of 14) over a 6-month period between 6m and 12m age. Undetectable DNA viral loads are shown as 5 HIV DNA cpm pbmc. **B.** Among all 17 children in whom undetectable DNA viral loads have been measured, a history of ART non-adherence was recorded in case report forms at similar levels in males and females who had achieved undetectable DNA viral loads (left panel). However, whereas viral suppression (plasma HIV RNA<20 c/ml) was maintained at similar levels in males and females in whom there was no history of ART non-adherence, where there was a history of ART non-adherence, all 4 females showed viral rebound while all 3 males maintained viral suppression ($p=0.029$, two-sided Fisher's Exact test). For Supplementary Figure 3B, source data are provided as a Source Data file.
