# Supplementary material for: Sex-specific innate immune selection of HIV-1 in utero is associated with increased female susceptibility to infection
Source: Nat Commun. 2020 Apr 14;11:1767. doi: 10.1038/s41467-020-15632-y (PMC7156749; doi:10.1038/s41467-020-15632-y)
Supplement: Supplementary file 3 — Reporting Summary [file 41467_2020_15632_MOESM3_ESM.pdf]

nature research

Corresponding author(s): Philip Goulder
Last updated by author(s): 27/02/20

Reporting Summary

Nature Research wishes to improve the reproducibility of the work that we publish. This form provides structure for consistency and transparency in reporting. For further information on Nature Research policies, see Authors & Referees and the Editorial Policy Checklist .

Statistics

For all statistical analyses, confirm that the following items are present in the figure legend, table legend, main text, or Methods section.

n/a	Confirmed	
☐	☒	The exact sample size (*n*) for each experimental group/condition, given as a discrete number and unit of measurement
☐	☒	A statement on whether measurements were taken from distinct samples or whether the same sample was measured repeatedly
☐	☒	The statistical test(s) used AND whether they are one- or two-sided<br>*Only common tests should be described solely by name; describe more complex techniques in the Methods section.*
☐	☒	A description of all covariates tested
☐	☒	A description of any assumptions or corrections, such as tests of normality and adjustment for multiple comparisons
☐	☒	A full description of the statistical parameters including central tendency (e.g. means) or other basic estimates (e.g. regression coefficient) AND variation (e.g. standard deviation) or associated estimates of uncertainty (e.g. confidence intervals)
☒	☐	For null hypothesis testing, the test statistic (e.g. *F*, *t*, *r*) with confidence intervals, effect sizes, degrees of freedom and *P* value noted<br>*Give P values as exact values whenever suitable.*
☒	☐	For Bayesian analysis, information on the choice of priors and Markov chain Monte Carlo settings
☒	☐	For hierarchical and complex designs, identification of the appropriate level for tests and full reporting of outcomes
☐	☒	Estimates of effect sizes (e.g. Cohen's *d*, Pearson's *r*), indicating how they were calculated

Our web collection on statistics for biologists contains articles on many of the points above.

Software and code

Policy information about availability of computer code

Data collection	Excel for Mac v16.3, DataFax v 2016.0.0 and DF Discover version 5.1.0 were used to collect data from the Ucwaningo Lwabantwana study
Data analysis	GraphPad Prism version 7.0b

For manuscripts utilizing custom algorithms or software that are central to the research but not yet described in published literature, software must be made available to editors/reviewers. We strongly encourage code deposition in a community repository (e.g. GitHub). See the Nature Research guidelines for submitting code & software for further information.

Data

Policy information about availability of data

All manuscripts must include a data availability statement. This statement should provide the following information, where applicable:

- Accession codes, unique identifiers, or web links for publicly available datasets
- A list of figures that have associated raw data
- A description of any restrictions on data availability

The data that support the findings in this study are available from the corresponding authors upon reasonable request.

Field-specific reporting

Please select the one below that is the best fit for your research. If you are not sure, read the appropriate sections before making your selection.

☒ Life sciences ☐ Behavioural & social sciences ☐ Ecological, evolutionary & environmental sciences

For a reference copy of the document with all sections, see nature.com/documents/nr-reporting-summary-flat.pdf

Life sciences study design

All studies must disclose on these points even when the disclosure is negative.

Sample size	Sample sizes were determined by considerations of sample availability, study resources, and previous paediatric cohort studies we have undertaken in South Africa (eg Muenchhoff et al Sci Transl Med 2016) where the numbers studied had allowed statistically significant immune predictors of disease outcomes to be made. No statistical methods were used to predetermine sample sizes.
Data exclusions	No data were excluded from analyses
Replication	Determination of viral replication capacities, interferon-resistance IC80, ddPCR determinations of HIV DNA load are all highly reproducible and are assays with a high degree of precision. In the case of the VRC and ddPCR assays these are performed in triplicate. In each case the reproducibility of the data from these assays has been demonstrated by repeat testing on a subset of the samples
Randomization	All mothers and infants were from the same ethnic population (South Africa).
Blinding	All the analyses were done blinded to sex of the subject.

Reporting for specific materials, systems and methods

We require information from authors about some types of materials, experimental systems and methods used in many studies. Here, indicate whether each material, system or method listed is relevant to your study. If you are not sure if a list item applies to your research, read the appropriate section before selecting a response.

Materials & experimental systems

n/a	Involved in the study	
☐	☒	Antibodies
☐	☒	Eukaryotic cell lines
☒	☐	Palaeontology
☒	☐	Animals and other organisms
☐	☒	Human research participants
☒	☐	Clinical data

Methods

n/a	Involved in the study	
☒	☐	ChIP-seq
☐	☒	Flow cytometry
☒	☐	MRI-based neuroimaging

Antibodies

Antibodies used	CD3 BV570 Biolegend, Cat no. 300436, clone UCHT1, Lot B215182; CD4 BV605 BD Biosciences Cat no. 562658, Clone RPA-T4 Lot 8004660; CD8 BV650 BD Biosciences Cat no. 563821, Clone RPA-T8, Lot no. 7240595; PD-1 PE-eflour 610 Invitrogen Cat no. 61-2799-42, Clone eBioJ105 Lot no. 1979718; HLA-DR APC BD Biosciences Cat no. 565127 Clone G46-6, Lot no. 6204610; CCR5 PECy7 Biolegend Cat no. 359107, Clone J418F1, Lot no. B231442; CD45RA AlexaFluor 700 Biolegend Cat no. 304120, Clone HI100, Lot no. B257456; CCR7 Pacific Blue Biolegend Cat no. 353210, Clone G043H7 Lot no. B255058; Live/Dead Stain Kit APCCy7 Life Technologies Cat no. L10119 Lot no. 1683631
Validation	Antibodies used were developed for the specific applications, and QC testing was done on human samples by the suppliers. Optimal antibody concentrations for use were determined by titration, and empirical testing of antibody combinations was carried out to establish the individual flow cytometry panels.

Eukaryotic cell lines

Policy information about cell lines

Cell line source(s)	CEM-GXR cells were provided by collaborators Mark Brockman at Simon Fraser University, Canada.
Authentication	CD4 and CXCR4 expression on CEM-GXR cells was verified in-house by flow cytometry; and NL4-3 used as a positive control to assess cell permissiveness to virus infection.
Mycoplasma contamination	These were received mycoplasma, bacteria and fungi free; a new vial of cells was received and used. No in-house testing for mycoplasma contamination was done in addition.
Commonly misidentified lines (See ICLAC register)	No commonly misidentified cell line was used in this study.

Human research participants

Policy information about studies involving human research participants

Population characteristics	The mother-child study cohort enrolled in KwaZulu-Natal, South Africa, comprised 177 mother-child pairs. Of the 177 in utero infected infants, 111 were female and 66 were male. At the time of the analysis, the median age of the 177 infants was 20 months (IQR 14m-31m). At the child's birth, the mothers' median age was 25yrs (IQR 21-30yrs), median absolute CD4 count was 457 cells/ul (IQR 313-650), median CD4% was 24% (IQR 18-31%) and median plasma viral load was 12,000 HIV RNA copies/ml plasma (IQR 1400-210,000). Median time between date of birth of the child and the first positive HIV test in the mother was 131 days (IQR 18-1129 days).
Recruitment	This has been described in detail in the text in the section entitled "Study subjects".
Ethics oversight	The Biomedical Ethics Research Committee, University of KwaZulu-Natal and the Oxford Research Ethics Committee approved the studies.

Note that full information on the approval of the study protocol must also be provided in the manuscript.

Flow Cytometry

Plots

Confirm that:

- [ ] The axis labels state the marker and fluorochrome used (e.g. CD4-FITC).
- [ ] The axis scales are clearly visible. Include numbers along axes only for bottom left plot of group (a 'group' is an analysis of identical markers).
- [ ] All plots are contour plots with outliers or pseudocolor plots.
- [ ] A numerical value for number of cells or percentage (with statistics) is provided.

Methodology

Sample preparation	Cord blood PBMC was thawed, washed and rested in R10 media for 1 hour then stained with the anibody panel at room temperature for 20 minutes. Thereafter samples were washed and resuspended in 2% PFA and acquired on the flow cytometer. CEM-GXR cells were harvested daily, fixed in 2% PFA and acquired on the flow cytometer for quantification of GFP expression.
Instrument	BD LSR II
Software	FlowJo Software (Tree Star Inc., Ashland, OR).
Cell population abundance	Immunophenotyping was conducted using whole PBMC samples, no post-sorting of cells was involved.
Gating strategy	Singlets were identified and further gated as lymphocytes based on SSC-A FSC-H distribution. Live cells were then gated followed by distinct populations of CD3+ CD4+ T cells, and then on select combinations of markers identifying specific immunophenotype markers such as HLA-DR expression. The gating strategy utilized was based on FMO whereby CD3+ CD4+ T cells lacking HLA-DR antibody served as negative expression of HLA-DR.

- [ ] Tick this box to confirm that a figure exemplifying the gating strategy is provided in the Supplementary Information.
